# Supplementary figures and images for: A Keller-Segel model for C elegans L1 aggregation
Source: PLoS Comput Biol. 2021 Jul 29;17(7):e1009231. doi: 10.1371/journal.pcbi.1009231 (PMC8354456; doi:10.1371/journal.pcbi.1009231)

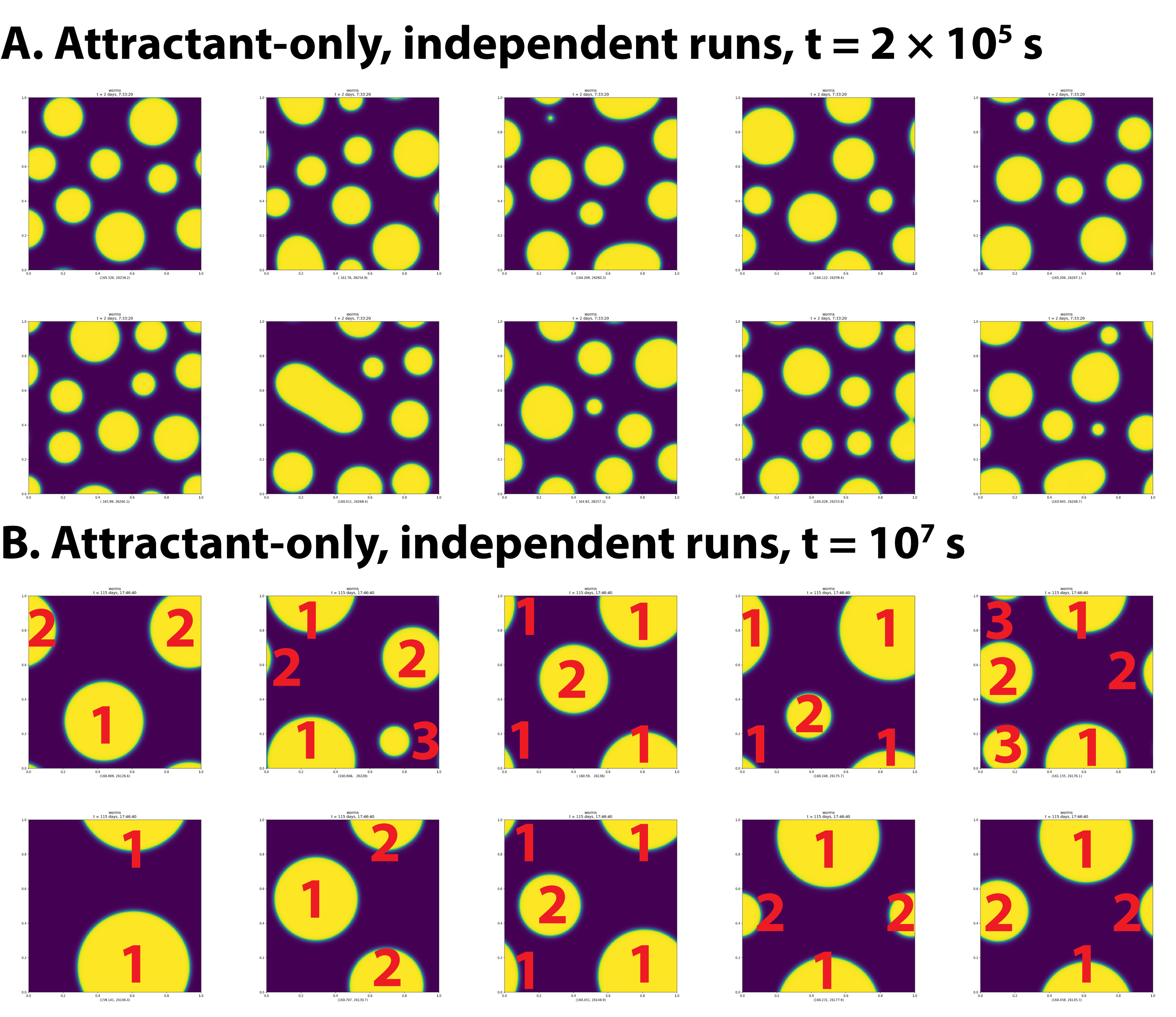

Supplement: S1 Fig — A. These ten images reproduce the numerical experiment of Fig 2C and 2D—simulation of the attractant-only model in two dimensions—but with different pseudorandom noise in the initial condition. Only worm density ρ(x, y) at t = 200 000 s (2 days, 7:33:20) is shown. B. Like A, but at t = 1 × 107 s (115 days, 17:46:40). These images correspond one-to-one to the images in A. The number of aggregates in these panels ranges from one to three, although a single aggregate may appear in as many as four pieces because of the periodic boundary conditions. To ease the identification of aggregates, the aggregate to which each piece belongs is identified by a red number. (TIF) [file pcbi.1009231.s001.tif]

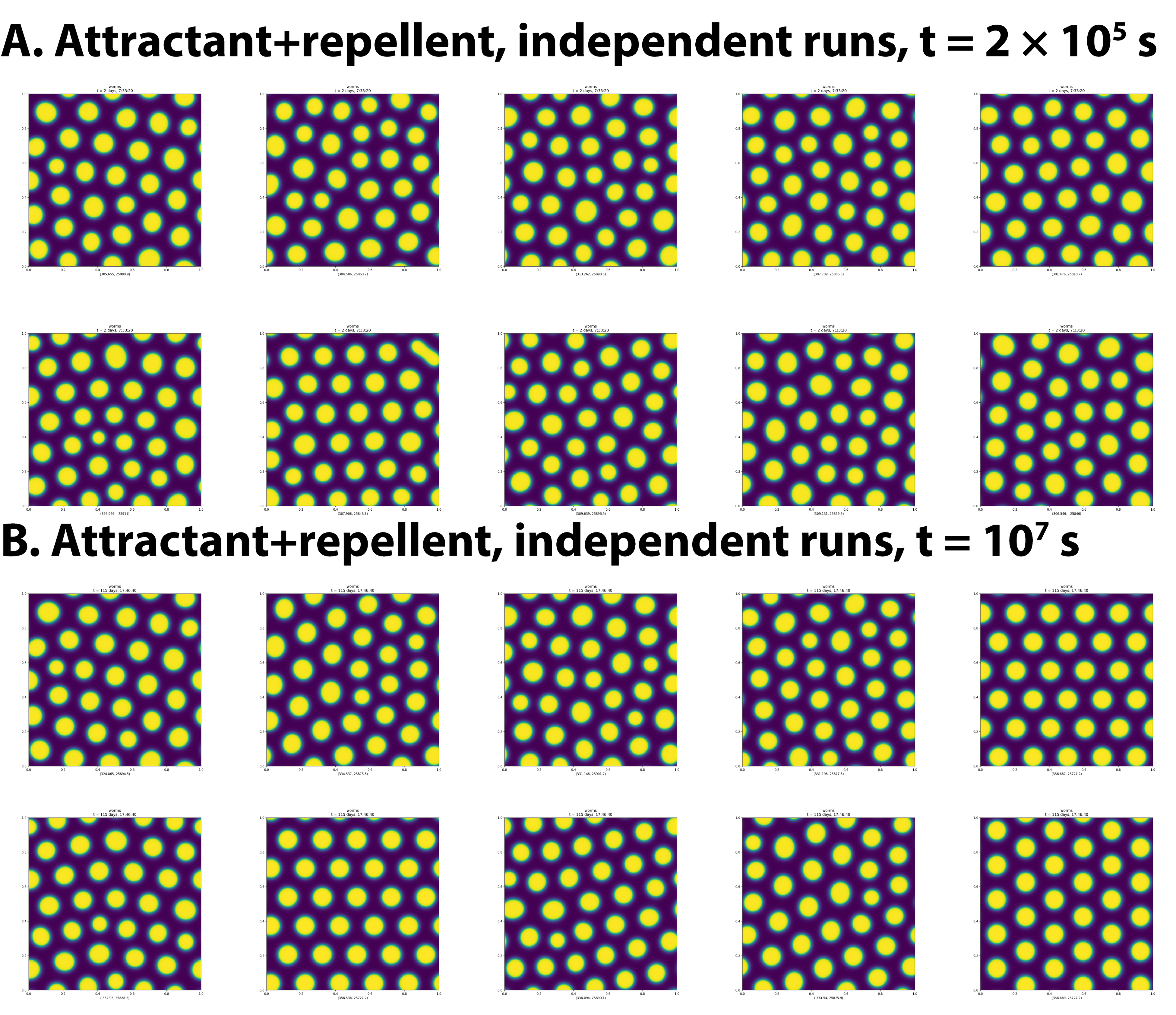

Supplement: S2 Fig — A. These ten images reproduce the numerical experiment of Fig 3D, 3E and 3F—simulation of the attractant+repellent model in two dimensions—but with different pseudorandom noise in the initial condition. Only worm density ρ(x, y) at t = 200 000 s (2 days, 7:33:20) is shown. B. Like A, but at t = 1 × 107 s (115 days, 17:46:40). These images correspond one-to-one to the images in A. (TIF) [file pcbi.1009231.s002.tif]

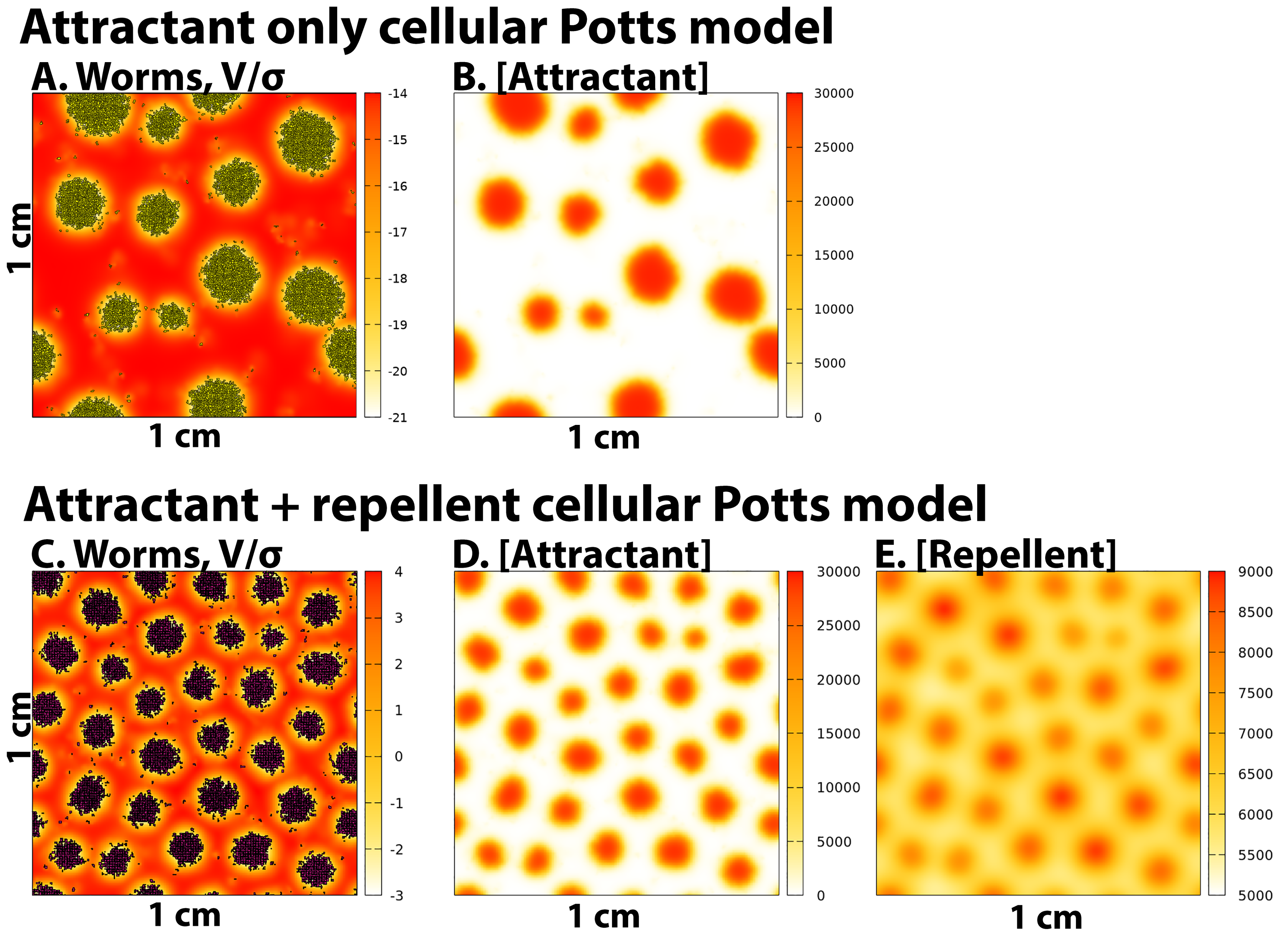

Supplement: S3 Fig — A, B These two images show the results of an individual-based cellular Potts model simulation of the Attractant-only model in two dimensions, and are meant to be compared to Fig 2C and 2D. D-F show the results of a cellular Potts model simulation of the attractant+repellent model in two dimensions and can be compared to Fig 3C, 3D and 3E. Because there is no simple relationship between the parameters of the PDE model and the cellular Potts model, we do not expect precise quantitative agreement, even on a statistical basis. (TIF) [file pcbi.1009231.s003.tif]
