# Supplementary material for: A Keller-Segel model for C elegans L1 aggregation
Source: PLoS Comput Biol. 2021 Jul 29;17(7):e1009231. doi: 10.1371/journal.pcbi.1009231 (PMC8354456; doi:10.1371/journal.pcbi.1009231)
Supplement: S1 Text — (PDF) [file pcbi.1009231.s004.pdf]

A Keller-Segel model for *C elegans* L1 aggregation

Supporting information S1 Text: Linear stability analysis of the attractant-only model

The attractant–only model (1, 2) shares with the original Keller-Segel system [1] the property of density-dependent instability. There is a uniform equilibrium,

$$\rho_{\mathrm{eq}}(t, \mathbf{x}) = \bar{\rho} \tag{S1-1}$$

$$U_{\mathrm{eq}}(t, \mathbf{x}) = \bar{U} \coloneqq \frac{s_a}{\gamma_a}\bar{\rho} \tag{S1-2}$$

Substituting these functions into (1, 2) shows that $\mathrm{d}\rho_{\mathrm{eq}}/\mathrm{d}t = \mathrm{d}U_{\mathrm{eq}}/\mathrm{d}t = 0$. Consider a population near this equilibrium, and let $\rho(t, \mathbf{x}) = \bar{\rho} + \delta\rho(t, \mathbf{x})$, $U(t, \mathbf{x}) = \bar{U} + \delta U(t, \mathbf{x})$. Because $(\bar{\rho}, \bar{U})$ is an equilibrium, $\rho_t(t, \mathbf{x}) = \delta\rho_t(t, \mathbf{x})$ and $U_t(t, \mathbf{x}) = \delta U_t(t, \mathbf{x})$.
Substituting into (1, 2),

$$\begin{aligned}
\delta\rho_t(t, \mathbf{x}) = {} & \boldsymbol{\nabla} \cdot \left(\delta\rho V_U'(U)\boldsymbol{\nabla}\bar{U} + \bar{\rho}V_U'(U)\boldsymbol{\nabla}\delta U\right) \\
& + \boldsymbol{\nabla} \cdot \left(\delta\rho V_\rho'(\rho)\boldsymbol{\nabla}\bar{\rho} + \bar{\rho}V_\rho'(\rho)\boldsymbol{\nabla}\delta\rho\right) \\
& + \boldsymbol{\nabla} \cdot \left(\delta\rho V_U'(U)\boldsymbol{\nabla}\delta U\right) \\
& + \sigma\nabla^2\bar{\rho} + \sigma\nabla^2\delta\rho
\end{aligned} \tag{S1-3}$$

$$\delta U_t(t, \mathbf{x}) = -\gamma\delta U + D\nabla^2\delta U + s\delta\rho \tag{S1-4}$$

$\boldsymbol{\nabla}\bar{U} = \boldsymbol{\nabla}\bar{\rho} = \nabla^2\bar{\rho} = 0$. Writing $V_U'(U) = V_U'(\bar{U}) + \mathscr{O}(\delta U)$, $V_\rho'(\rho) = V_\rho'(\bar{\rho}) + \mathscr{O}(\delta\rho)$ and ignoring second order terms we have, to first order, the linear vector PDE,

$$\frac{\partial}{\partial t}\begin{pmatrix}\delta\rho \\ \delta U\end{pmatrix} = \begin{pmatrix}(\sigma + V_\rho'(\bar{\rho}))\nabla^2 & V_U'(\bar{U})\bar{\rho}\nabla^2 \\ s & -\gamma + D\nabla^2\end{pmatrix}\begin{pmatrix}\delta\rho \\ \delta U\end{pmatrix} \tag{S1-5}$$

This is easily solved by separation of variables. It will be convenient to define $\sigma' = \sigma + V_\rho'(\bar{\rho})$. Since V_ρ is, by design, an increasing function, $V_\rho' > 0$ and $\sigma' > \sigma > 0$. For the parameters in Table 2, $V_\rho'(\bar{\rho}) \approx 0$ and $\sigma' \approx \sigma$. Now, eigenfunctions of (S1-5) are of the form

$$\begin{pmatrix} \delta\rho(t,\mathbf{x}) \\ \delta U(t,\mathbf{x}) \end{pmatrix} = \begin{pmatrix} \rho_{\mathbf{k}} e^{\lambda_{\mathbf{k}} t} e^{i\mathbf{k}\cdot\mathbf{x}} \\ U_{\mathbf{k}} e^{\lambda_{\mathbf{k}} t} e^{i\mathbf{k}\cdot\mathbf{x}} \end{pmatrix} \tag{S1-6}$$

$\mathbf{k}$ is a wavenumber vector, i.e., a vector of frequency in each spatial direction. Substituting into (S1-5) produces the 2×2 matrix eigenvalue problem,

$$\lambda_{\mathbf{k}} \begin{pmatrix} \rho_{\mathbf{k}} \\ U_{\mathbf{k}} \end{pmatrix} = \begin{pmatrix} -\sigma' k^2 & -V_U'(\bar{U})\bar{\rho}k^2 \\ s & -\gamma - Dk^2 \end{pmatrix} \begin{pmatrix} \rho_{\mathbf{k}} \\ U_{\mathbf{k}} \end{pmatrix} \tag{S1-7}$$

where $k^2 \coloneqq \|\mathbf{k}\|^2$. Solutions of the linearized system (S1-7) are linear combinations of functions (S1-6) where $(\rho_{\mathbf{k}}, U_{\mathbf{k}})^\intercal$ is an eigenvector of the matrix in (S1-7). If, for every $\mathbf{k}$, $\mathrm{Re}(\lambda_{\mathbf{k}}) < 0$ then any small fluctuation away from the uniform equilibrium will die away, and the uniform equilibrium will be stable. If, however, there exists a $\mathbf{k}$ such that the matrix has an eigenvalue with positive real part, then the uniform equilibrium is unstable. The sum of the two eigenvalues, the trace of the matrix, is negative, $-\sigma' k^2 - \gamma - Dk^2 < 0$, so the only possible way to have an eigenvalue with positive real part is if both eigenvalues are real, one positive and one negative. Thus, first-order instability is expected if and only if the determinant is negative,

$$\begin{vmatrix} -\sigma' k^2 & -V_U'(\bar{U})\bar{\rho}k^2 \\ s & -\gamma - Dk^2 \end{vmatrix} < 0 \tag{S1-8}$$

$$D\sigma' k^4 + \left(\gamma\sigma' + V_U'(\bar{U})s\bar{\rho}\right)k^2 < 0 \tag{S1-9}$$

Inequality (S1-9) can hold only if $\gamma\sigma' + V_U'(\bar{U})s\bar{\rho} < 0$. Since, as mentioned above, V_U is a decreasing function of attractant concentration, $V_U'(\bar{U}) < 0$. If the condition $\gamma\sigma' + V_U'(\bar{U})s\bar{\rho} < 0$ holds, then for some small enough k^2, the negative k^2 term will dominate the positive k^4 term and the determinant will be negative. Thus, first-order instability occurs if and only if $\bar{\rho} > -\frac{\gamma\sigma'}{sV_U'(\bar{U})}$. Since $\bar{U} \coloneqq s\bar{\rho}/\gamma$ depends on $\bar{\rho}$, it is not a

foregone conclusion that instability is possible. Neglecting V'_ρ and with V_U as in (7), the instability condition reduces to

$$\bar{\rho} > \frac{\alpha\gamma\sigma}{s(\beta - \sigma)} > 0 \tag{S1-10}$$

Instability is possible if $\beta > \sigma$. By design, the instability condition is $\bar{\rho} >$1500 $\mathrm{cm}^{-\mathrm{d}}$ with the parameter values in Table 2.

References

References

1. Keller EF, Segel LA. Initiation of slime mold aggregation viewed as an instability. Journal of Theoretical Biology. 1970;26(3):399–415. doi:10.1016/0022-5193(70)90092-5.
