# Supplementary material for: A Keller-Segel model for C elegans L1 aggregation
Source: PLoS Comput Biol. 2021 Jul 29;17(7):e1009231. doi: 10.1371/journal.pcbi.1009231 (PMC8354456; doi:10.1371/journal.pcbi.1009231)
Supplement: S2 Text — (PDF) [file pcbi.1009231.s005.pdf]

Supporting File 2: Linear stability analysis of the attractant+repellent model

For simplicity, we assume $V_\rho = 0$ in the following analysis. With the parameter values in Table 2, this is very close to true in the vicinity of the instability threshold. Linearization of the PDE system (2, 13, 14) around a uniform equilibrium at $\rho_{\mathrm{eq}}(t, \mathbf{x}) = \bar{\rho}$, $U_{i,\mathrm{eq}}(t, \mathbf{x}) = \bar{U}_i = s_i \bar{\rho}/\gamma_i$ (for $i \in a, r$) produces the following linear PDE system

$$\frac{\mathrm{d}}{\mathrm{d}t}\begin{pmatrix}\delta\rho \\ \delta U_a \\ \delta U_r\end{pmatrix} = \begin{pmatrix}\sigma\nabla^2 & \bar{\rho}V'_{U_a}(\bar{U_a})\nabla^2 & \bar{\rho}V'_{U_r}(\bar{U_r})\nabla^2 \\ s_a & -\gamma_a + D_a\nabla^2 & 0 \\ s_r & 0 & -\gamma_r + D_r\nabla^2\end{pmatrix}\begin{pmatrix}\delta\rho \\ \delta U_a \\ \delta U_r\end{pmatrix} \tag{S2-1}$$

The ansatz

$$\begin{pmatrix}\delta\rho \\ \delta U_a \\ \delta U_r\end{pmatrix} = \begin{pmatrix}\rho_{\mathbf{k}} \\ U_{a\mathbf{k}} \\ U_{r\mathbf{k}}\end{pmatrix} e^{\lambda_{\mathbf{k}} t} e^{i\mathbf{k}\cdot\mathbf{x}} \tag{S2-2}$$

yields the eigenvalue problem

$$\lambda_{\mathbf{k}} \begin{pmatrix} \rho_{\mathbf{k}} \\ U_{a\mathbf{k}} \\ U_{r\mathbf{k}} \end{pmatrix} = \begin{pmatrix} -\sigma k^2 & -\bar{\rho} V'_{U_a}(\bar{U_a}) k^2 & -\bar{\rho} V'_{U_r}(\bar{U_r}) k^2 \\ s_a & -\gamma_a - D_a k^2 & 0 \\ s_r & 0 & -\gamma_r - D_r k^2 \end{pmatrix} \begin{pmatrix} \rho_{\mathbf{k}} \\ U_{a\mathbf{k}} \\ U_{r\mathbf{k}} \end{pmatrix} \tag{S2-3}$$

$$= \begin{pmatrix} -\sigma k^2 & -\bar{\rho} k^2 (\mathbf{V}'(\bar{\mathbf{U}}))^\intercal \\ \bar{\rho}\mathbf{s} & -\mathbf{\Gamma} - \mathbf{D} k^2 \end{pmatrix} \begin{pmatrix} \rho_{\mathbf{k}} \\ \mathbf{U}_{\mathbf{k}} \end{pmatrix} \tag{S2-4}$$

$$= -\mathbf{N}(\bar{\rho}, k^2) \begin{pmatrix} \rho_{\mathbf{k}} \\ \mathbf{U}_{\mathbf{k}} \end{pmatrix} \tag{S2-5}$$

In (S2-4), the matrix is in a block form that can be extended easily to any number of signals, with

$$\mathbf{s} = \begin{pmatrix} s_a \\ s_r \end{pmatrix} \tag{S2-6}$$

$$\mathbf{U}_{\mathbf{k}} = \begin{pmatrix} U_{a\mathbf{k}} \\ U_{r\mathbf{k}} \end{pmatrix} \tag{S2-7}$$

$$\mathbf{V}'(\bar{\mathbf{U}}) = \begin{pmatrix} V'_{U_a}(\bar{U_a}) \\ V'_{U_r}(\bar{U_r}) \end{pmatrix} \tag{S2-8}$$

$$\mathbf{\Gamma} = \begin{pmatrix} \gamma_a & 0 \\ 0 & \gamma_r \end{pmatrix} \tag{S2-9}$$

$$\mathbf{D} = \begin{pmatrix} D_a & 0 \\ 0 & D_r \end{pmatrix} \tag{S2-10}$$

In (S2-5), $\mathbf{N}(\bar{\rho}, k^2)$ is defined as the negative of the matrix in (S2-4). (We define $\mathbf{N}$ as the negative to avoid an inconvenient factor of $(-1)^{1+n_{\text{signals}}}$ in the determinant we are about to calculate.) The uniform equilibrium is unstable at mean density $\bar{\rho}$ if for some k, $\mathbf{N}(\bar{\rho}, k^2)$ has an eigenvalue with negative real part. If $\left|\mathbf{N}(\bar{\rho}, k^2)\right| < 0$, the equilibrium is certainly unstable. This leads to the following criterion for instability

$$-\sigma > \bar{\rho}(\mathbf{V}'(\bar{\mathbf{U}}))^{\intercal}(\mathbf{\Gamma} + \mathbf{D}k^2)^{-1}\mathbf{s} \tag{S2-11}$$

$$= \bar{\rho} \sum_{i\in\{a,r\}} \frac{\mathbf{V'}_{U_i}(\bar{U_i})s_i}{\gamma_i + D_i k^2} \tag{S2-12}$$

$$= \bar{\rho}\left(\frac{\mathbf{V'}_{U_a}(\bar{U_a})s_a}{\gamma_a + D_a k^2} + \frac{\mathbf{V'}_{U_r}(\bar{U_r})s_r}{\gamma_r + D_r k^2}\right) \tag{S2-13}$$

It is possible to choose parameter values so that this criterion predicts instability with a nontrivial minimum wavenumber (and therefore finite maximum scale). How does this work? Remember that $V'_{U_r} > 0$ because it is a repellent and $V'_{U_a} < 0$ because it is an attractant. Thus the two terms in (S2-13) are opposite in sign. Also, $\gamma_r < \gamma_a$ and $D_r > D_a$, because the repellent is a longer-range signal than the attractant. Thus, at low k, if the relative magnitudes of $V'_{U_r}s_r$ and $V'_{U_a}s_a$ are appropriately adjusted (by evolution or the modeler), the sum in (S2-13) is positive and the uniform equilibrium is stable to perturbations of small wavenumber = large scale. As k rises the $D_r k^2$ factor in the denominator of the repellent term makes the positive term small compared to the negative attractant term. The sum in parentheses can become negative, and if $\bar{\rho}$ is large enough, the right-hand-side drops below $-\sigma$, and instability to perturbations of intermediate wavenumber = medium scale results. For large k the right-hand-side approaches zero because of the Dk^2 factors in both denominators. The uniform equilibrium is thus stable to perturbations of large wavenumber = small scale. It is therefore possible for an attractant+repellent Keller-Segel model to have a finite natural scale.

Based on calculations of this sort we chose $\beta_r = -\beta_a = -2\sigma = -5.56 \times 10^{-4}\,\mathrm{cm}^2\mathrm{s}^{-1}$. Attractant parameters remained as in Table 2. The addition of a repellent increases the threshold for instability, but the uniform equilibrium is still predicted to be unstable at $\bar{\rho} = 9000\,\mathrm{cm}^{-}\mathrm{d}$.
