# Supplementary material for: A Keller-Segel model for C elegans L1 aggregation
Source: PLoS Comput Biol. 2021 Jul 29;17(7):e1009231. doi: 10.1371/journal.pcbi.1009231 (PMC8354456; doi:10.1371/journal.pcbi.1009231)
Supplement: S3 Text — (PDF) [file pcbi.1009231.s006.pdf]

Supporting Information S3 Text: Convergence tests

To test whether the numerical solution of the worm system PDEs (2, 13, 14) approximates the correct solution, we compared numerical solutions of the attractant+repellent system to an analytical solution of the linearized PDE system (S2-1). In one dimension, for $x \in \Omega = [0, 1]$,

$$\delta\rho(t, x) \coloneqq a_\rho e^{\lambda t} \sin(\phi + 2\pi k_0 x) \tag{S3-1}$$

$$\rho_{\text{lin}}(t, x) = \bar{\rho} + \delta\rho(t, x) \tag{S3-2}$$

$$U_{a,\text{lin}}(t, x) = \frac{s_a}{\gamma_a}\bar{\rho} + U_{a,k_0}\delta\rho(t, x) \tag{S3-3}$$

$$U_{r,\text{lin}}(t, x) = \frac{s_r}{\gamma_r}\bar{\rho} + U_{r,k_0}\delta\rho(t, x) \tag{S3-4}$$

In two dimensions, for $(x, y) \in \Omega = [0, \sqrt{3}/2] \times [0, 1/2]$,

$$\delta\rho(t, x, y) \coloneqq a_\rho e^{\lambda t}\bigg(\frac{1}{3}\big(\cos(2\pi k_0 y) + \cos(2\pi(k_{x,0}x - k_{y,0}y)) + \cos(2\pi(k_{x,0}x + k_{y,0}y))\big)\bigg) \tag{S3-5}$$

$$\rho_{\text{lin}}(t, x, y) = \bar{\rho} + \delta\rho(t, x, y) \tag{S3-6}$$

$$U_{a,\text{lin}}(t, x, y) = \frac{s_a}{\gamma_a}\bar{\rho} + U_{a,k_0}\delta\rho(t, x, y) \tag{S3-7}$$

$$U_{r,\text{lin}}(t, x, y) = \frac{s_r}{\gamma_r}\bar{\rho} + U_{r,k_0}\delta\rho(t, x, y) \tag{S3-8}$$

Here $\phi, a_\rho \in \mathbb{R}$ and $k_0 \in 2\mathbb{Z}$ are parameters that can be freely chosen. We chose $a_\rho = 1$ and $\phi = \pi/2$. We chose $k_0 = 4$ to produce a substantial positive growth rate. For the

two-dimensional case, we chose $k_{x,0} = k_0\sqrt{3}/2$ and $k_{y,0} = k_0/2$ to produce hexagonal symmetry. λ is the positive eigenvalue of matrix $-\mathbf{N}(\bar{\rho}, k_0^2)$ (S2-5), with corresponding eigenvector $(1, U_{ak_0}, U_{rk_0})^\intercal$ (as in (S2-2), but normalized so that $\rho_{k_0} = 1$). Numerical values $\lambda \approx 0.000\,955\,\mathrm{s}^{-1}$, $U_{ak_0} \approx 0.863$, $U_{rk_0} \approx 0.121$ were estimated to 15-digit precision by numerical diagonalization of the computed matrix $-\mathbf{N}(\bar{\rho}, k_0^2)$.

Functions $(\rho_{\mathrm{lin}}(t,x), U_{a,\mathrm{lin}}(t,x), U_{r,\mathrm{lin}}(t,x))^\intercal$ are of course not an exact solution of the full nonlinear PDEs (2, 13, 14). To produce a closely related system with this exact solution for convergence testing, we modified the ρ PDE (2) by addition of a source term $S(t,\mathbf{x})$.

$$\dot{\rho} = \nabla\cdot\left(\rho\nabla(V_{U_a}(U_a) + V_{U_r}(U_r) + V_\rho(\rho) + \sigma\log\rho)\right) + S(t,\mathbf{x}) \tag{S3-9}$$

$$\begin{aligned} S(t,\mathbf{x}) = \frac{\partial\rho_{\mathrm{lin}}(t,\mathbf{x})}{\partial t} - \boldsymbol{\nabla}\cdot\big(&\rho_{\mathrm{lin}}(t,\mathbf{x}) \\ &\boldsymbol{\nabla}\big(V_{U_a}(U_{a,\mathrm{lin}}(t,\mathbf{x})) + V_{U_r}(U_{r,\mathrm{lin}}(t,\mathbf{x})) \\ &+ V_\rho(\rho_{\mathrm{lin}}(t,\mathbf{x})) \\ &+ \sigma\log\rho_{\mathrm{lin}}(t,\mathbf{x})\big)\big) \end{aligned} \tag{S3-10}$$

It was unnecessary to modify the U_a and U_r PDEs since they are linear. Source function (S3-10) was computed symbolically from linear solutions (S3-2-S3-4) or (S3-6-S3-8) and converted to `sympy` expressions with Mathematica [1]. `sympy` is a computer algebra package for the programming language `python`.

References

References

1. Wolfram Research I. Mathematica; 2019.

A. Time step size series, one dimensional

Δt (s)	$\|error\|$ (cm^{-1}) L^2	L^∞	convergence rate L^2	L^∞
4	0.0100	0.0220	−0.064	−0.054
8	0.0096	0.0212	0.210	0.237
16	0.0111	0.0250	1.16	1.14
32	0.0247	0.0552	2.28	2.26
64	0.120	0.265	2.95	2.93
128	0.930	2.02	3.08	3.07
256	7.88	17.0		

B. Spatial point distance series, one dimensional

Δx (cm)	$\|error\|$ (cm^{-1}) L^2	L^∞	convergence rate L^2	L^∞
1/1024	0.0086	0.0196	0.216	0.162
1/512	0.0100	0.0220	1.28	1.22
1/256	0.0244	0.0514	3.43	3.33
1/128	0.263	0.516	3.95	3.95
1/64	4.07	7.97		

Table S3-1. Convergence test results, one spatial dimensional
Eqs. (S3-9, 13, 14) were solved numerically from $t = 0\,\text{s}$ to $8192\,\text{s}$ on $x \in \Omega = [0, 1]$. In this time the amplitude of the sinusoid $\delta\rho$ (S3-1) grew from $a_\rho = 1$ to $a_\rho e^{8192\lambda} \approx 2505$. **A** shows the results of varying the time step size from 4 to 256 s (with a fixed spatial point distance of Δx =1/512 cm). **B** shows the results of varying the spatial point distance from 1/1024 to 1/64 cm (with a fixed time step of 4 s). The error in ρ at the final time point was calculated as the difference between the numerical result and exact result (S3-2). L^2 and L^∞ norms of the error are tabulated. Convergence rate is calculated between consecutive rows as $\log(\|\text{error}_1\|/\|\text{error}_2\|)/\log(h_1/h_2)$, with h being either Δt or Δx, as appropriate. The mean of ρ was $9000\,\text{cm}^{-1}$ in all cases. Thus the relative error is about 1/9000 times the error shown—e.g. $0.0100/9000 \approx 1.1 \times 10^{-6}$ for $\Delta t = 4\,\text{s}$, $\Delta x = 1/512$ cm in one dimension. Errors in U_a and U_r (not shown) were smaller but otherwise behaved similarly. All numerical solutions used the PETSc Rosenbrock-W solver `ra34pw2` (nominally an order 3 method), and fourth-order approximations for the spatial derivatives.

A. Time step size series, two dimensional

	$\|error\|$ (cm^{-1})		convergence rate	
Δt (s)	L^2	L^∞	L^2	L^∞
4	0.0072	0.0469	2.01	2.20
8	0.0290	0.215	0.692	0.627
16	0.0468	0.332	−1.57	−2.21
32	0.0158	0.0716	3.40	3.48
64	0.167	0.800	1.53	1.28
128	0.483	1.94	3.14	3.21
256	4.27	18.0		

B. Spatial point distance series, two dimensional

	$\|error\|$ (cm^{-1})		convergence rate	
Δx (cm)	L^2	L^∞	L^2	L^∞
$\sqrt{3}/2048$	0.0246	0.178	−1.77	−1.92
$\sqrt{3}/1024$	0.0072	0.0469	0.103	−0.172
$\sqrt{3}/512$	0.0077	0.0416	2.84	2.26
$\sqrt{3}/256$	0.0555	0.199	3.92	3.86
$\sqrt{3}/128$	0.838	2.88		

Table S3-2. Convergence test results, two spatial dimensions
Eqs. (S3-9, 13, 14) were solved numerically from $t = 0\,\text{s}$ to $8192\,\text{s}$ on $(x, y) \in \Omega = [0, \sqrt{3}/2] \times [0, 1/2]$. In this time the amplitude of the sinusoid $\delta\rho$ (S3-5) grew from $a_\rho = 1$ to $a_\rho e^{8192\lambda} \approx 2505$. **A** shows the results of varying the time step size from 4 to 256 s (with a fixed spatial point distance of $\Delta x = \sqrt{3}/1024$ cm). **B** shows the results of varying the spatial point distance from $\sqrt{3}/2048$ to $\sqrt{3}/128$ cm (with a fixed time step of $4\,\text{s}$). In all cases $\Delta y = \Delta x \times 64\sqrt{3}/111$. Errors and convergence rates calculated as in Table S3-1.
